# Dehydration stress memory genes of *Zea mays*; comparison with *Arabidopsis thaliana*

**DOI:** 10.1186/1471-2229-14-141

**Published:** 2014-05-22

**Authors:** Yong Ding, Laetitia Virlouvet, Ning Liu, Jean-Jack Riethoven, Michael Fromm, Zoya Avramova

**Affiliations:** 1 University of Science & Technology of China, 443 Huangshang Road, Hefei, Anhui 230027, China; 2 University of Nebraska School of Biological Sciences, 1901 Vine Street, Lincoln 68588, USA; 3 University of Nebraska Center for Biotechnology and Center for Plant Science Innovation, 1901 Vine Street, Lincoln 68588, USA

## Abstract

**Background:**

Pre-exposing plants to diverse abiotic stresses may alter their physiological and transcriptional responses to a subsequent stress, suggesting a form of “stress memory”. Arabidopsis thaliana plants that have experienced multiple exposures to dehydration stress display transcriptional behavior suggesting “memory” from an earlier stress. Genes that respond to a first stress by up-regulating or down-regulating their transcription but in a subsequent stress provide a significantly different response define the ‘memory genes’ category. Genes responding similarly to each stress form the ‘non-memory’ category. It is unknown whether such memory responses exists in other Angiosperm lineages and whether memory is an evolutionarily conserved response to repeated dehydration stresses.

**Results:**

Here, we determine the transcriptional responses of maize (*Zea mays* L.) plants that have experienced repeated exposures to dehydration stress in comparison with plants encountering the stress for the first time. Four distinct transcription memory response patterns similar to those displayed by *A. thaliana* were revealed. The most important contribution is the evidence that monocot and eudicot plants, two lineages that have diverged 140 to 200 M years ago, display similar abilities to ‘remember’ a dehydration stress and to modify their transcriptional responses, accordingly. The highly sensitive RNA-Seq analyses allowed to identify genes that function similarly in the two lineages, as well as genes that function in species-specific ways. Memory transcription patterns indicate that the transcriptional behavior of responding genes under repeated stresses is different from the behavior during an initial dehydration stress, suggesting that stress memory is a complex phenotype resulting from coordinated responses of multiple signaling pathways.

**Conclusions:**

Structurally related genes displaying the same memory responses in the two species would suggest conservation of the genes’ memory during the evolution of plants’ dehydration stress response systems. On the other hand, divergent transcription memory responses by genes encoding similar functions would suggest occurrence of species-specific memory responses. The results provide novel insights into our current knowledge of how plants respond to multiple dehydration stresses, as compared to a single exposure, and may serve as a reference platform to study the functions of memory genes in adaptive responses to water deficit in monocot and eudicot plants.

## Background

Water deficit affects a broad range of plant functions, including growth, photosynthesis, metabolic pathways, and if severe enough, can cause tissue damage and death [1]. Plants respond to dehydration stress through physiological adjustments presumably regulated by the expression of specific genes involved in the dehydration stress response [2,3]. Pre-exposure to diverse types of stresses, including dehydration stress, may alter subsequent responses, suggesting a form of “stress memory” [4-7]. *A. thaliana* plants that have undergone one or more alternating cycles of dehydration stress treatments following periods of full watered recovery (“trained” plants) retain a memory of an earlier dehydration stress as evidenced by the greater ability to retain leaf relative water content (RWC) than plants experiencing dehydration stress for the first time (“untrained” plants) [8]. Trained plants responded differently also at the transcription level as several ABA-inducible genes displayed higher transcription rates and transcript levels during a subsequent exposure, a behavior consistent with transcriptional memory [8]. In a recent whole-genome transcriptome analysis of *A. thaliana*, we found an unexpected diversity of transcriptional memory patterns during repeated stress treatments [9].

Arabidopsis is a model system for plant biology research and for suggesting methodologies to impact processes of importance for agriculture [10]. To find out whether dehydration stress memory exists in Angiosperm lineages other than Arabidopsis, we conducted whole-genome transcriptome analysis of repetitively stressed maize (*Zea mays* L.) plants. Identification of homologous genes that display the same memory-type responses would suggest evolutionarily conserved memory responses in two lineages that have diverged over 140 to 200 M years [11]. On the other hand, non-memory or divergent memory responses by homologous genes would illustrate diversification and specialization of dehydration stress responses and/or of the functions of these genes during their evolution.

The experimental repetitive-dehydration stress system involves exposing seedlings to successive cycles of air drying, overnight watered recovery, followed by a new exposure to dehydrating conditions [8]. This procedure has similarities to the daily oscillation in water potential that occurs in plants growing in soil with decreasing water availability during drought conditions [12]. Here, we analyze the transcriptional behavior of genes involved in the dehydration stress response to assess the conserved and divergent dehydration stress memory responses of structurally related genes in maize and in Arabidopsis, and compare potential cellular functions encoded by dehydration memory genes in the two species.

## Methods

### Plant growth and treatments

*Zea mays* L. (cultivar B73) seedlings were grown in soil in greenhouse with regular watering. Two-week old seedlings were removed from soil and acclimated overnight in trays with their roots in water. The next morning the seedlings were exposed to air drying for 2 h at 22°C (first dehydration stress, S1) followed by a period of full re-hydration recovery for 22 h at 22°C, as described earlier [8]. Repeated dehydration stresses were performed following the same alternating cycles of exposure to air drying/overnight watered recovery. Leaves from non-stressed watered (W) plants, from plants that have experienced a single stress exposure (S1) and three stress exposures (S3) were analyzed. Seven to ten individual plants were used in each treatment point and two independent biological samples were used in the RNA-seq analyses. The relative water content (RWC) was measured in leaves detached from plants and immediately weighed to determine their FW (fresh weight). The same leaves were submerged in deionized water for 24 h, blotted dry and weighed to determine their TW (turgid weight). DW (dry weight) measurements were taken after the leaves were oven-dried (65°C) in brown paper bags for 24 h. The RWC value was calculated using the formula: RWC (%) = ((FW - DW)/(TW - DW)) × 100% [13].

### RNA extraction and RNA-Seq library construction

Leaf tissues were collected and immediately frozen in liquid nitrogen. Total RNA was extracted with Trizol (Invitrogen Inc. Carlsbad, CA, USA), treated with DNase I (Qiagen, Valencia, CA), and purified using Qiagen RNeasy. RNA integrity was confirmed on a Bioanalyzer 2100 using a Nano 6000 LabChip (Agilent Technologies, Santa Clara, CA). Complementary DNA sequencing library was prepared from the total RNA using the mRNA-Seq Sample Preparation Kit (Illumina, San Diego, CA). The resultant cDNA libraries were size-fractionated on an agarose gel, 200 bp fragments excised, and amplified by 15 cycles of polymerase chain reaction. Clusters were generated from the cDNA sequencing library on the surface of a flow cell in the Cluster Station (Illumina) by so-called bridge amplification. Replicates for the watered, S1 and S3 sample libraries were each run on a single lane in a flow cell on an Illumina GAIIx at the Genomics Core Facility at the University of Nebraska-Lincoln.

### Reverse transcription and real-time PCR

Total RNA isolation and reverse transcription with oligo(dT) (18418–012, Invitrogen) were performed as described previously [14]. The amounts of individual genes were measured with gene-specific primers by real-time PCR analysis with a iCycler iQ real-time PCR Instrument (Bio-Rad) and SYBR Green mixture (Bio-Rad). The relative expression or amount of specific genes was quantitated with the 2^-ΔΔ*C*t^ calculation [15], according to the manufacturer’s software (Bio-Rad), where the reference gene was ubiquitin. Primers used in real-time RT-PCR are in Additional file 1.

### Bioinformatics analysis

Transcriptome sequencing of the watered, S1, and S3 samples yielded a total of 76.0, 74.6, and 71.7 million reads, respectively, summed over the two biological replicates per sample (Additional file 2). The read length for each sample’s first replicate is 75 bases, and for the second replicate is 76 bases. To determine the quality of the replicates we performed a least-square simple linear regression for each of the three samples. We calculated the *R*^*2*^ statistic (0.94 ≤ *R*^*2*^ ≤ 0.98) and slope (0.81 ≤ *b* ≤ 1.00), which provide measures of goodness-of-fit and correlation, respectively, using the *regress* function in MATLAB® (version 8.1.0.604 [R2013a]; The MathWorks™, Additional file 2). For use in all further analyses, the Zea mays genome (Maize Golden Path B73 RefGen_v2 assembly), gene models (version 5b, Filtered Gene Set FGS), and functional annotations were downloaded from the MaizeGDB [16].

The *bowtie* (version 2.1.0; [16]) and *tophat* (version 2.0.8; [17]) packages were used with default parameters to map the RNA sequence reads from watered, S1, and S3 to the genome and to determine the expression quantity of known transcripts in each sample. The *cuffdiff* tool from the cufflinks package (version 2.0.2; [18]) was run with default parameters to calculate expression changes and associated q-values (False Discovery Rate adjusted p-values) for each gene, between the samples S1 and water, and S3 and S1.

We further classify genes as being significantly differentially expressed when all three of the following conditions are met: *q* ≤ 0.05; | log_2_(fold change) | ≥ 1; and the FPKM-normalized expression value of at least one sample out of the two needs to be larger than the 25th percentile. The output files of *cuffdiff* are further annotated (in-house Perl scripts) by adding gene functional descriptions from the B73 RefGen_v2 annotations. Since only 7164 out of 39,635 genes had annotations other than “hypothetical” or “putative” protein, we decided to use protein homologies with a well-studied model organism, namely *Arabidopsis thaliana*, to infer gene descriptions and GO classifications to each *Zea mays* gene. *Arabidopsis* proteins and Gene Ontology assignments were downloaded from the Arabidopsis Information Resource (release TAIR10, [19]). For each *Zea mays* protein, we used *BLASTP* (version 2.2.28+; [20]) with an e-value of 10^-3^ to get a list of hits against *Arabidopsis* proteins; then we sort the hits based on ascending e-value, descending length, and descending percentage of identity (pid). From that sorted list, we take all matches up to the point where the e-value is 10^10^ times worse than the best hit (e.g. 10^-63^ to 10^-53^). Using such a range of e-values allows us to narrowly sample the *Arabidopsis* proteins with the closest homologies. From these, we summarize a functional description using the top 3 longest common substrings from the TAIR10 descriptions, and to create for each GO domain (cellular component, molecular function, and biological process) a list of the top 10 occurring terms. We used the new inferred description if the *Zea mays* annotation was absent, and we always assigned GO terms via this methodology to the *Zea mays* proteins.

All annotation thus obtained is merged into a master file containing all data for S1 versus water and S3 versus S1 gene expression profiles (Additional file 3). From that master file we determined the 2062 significant drought-responsive genes (S1 versus water), and using that initial set we then looked at those with significantly different responses in S3 versus S1 (816 genes). We assigned simple classifications to the types of response during the first stress (S1) (+or -) indicating transcript levels higher, or lower, than watered (W) levels. For the subsequent (S3) stress the signs (+, -, or =) indicate transcripts at levels higher, lower, or the same, as the levels in S1. Accordingly, response genes were combined into six classes: [++], [--], [+-], [-+], [+=], and [-=], the first four representing the memory categories, the latter two representing the non-memory classes (Additional file 4). Two additional classes [=/+] and [=/-] contain genes that did not change significantly expression in S1 compared to pre-stressed levels in W (according to the three criteria for significance above). These genes significantly changed transcription in S3 defining a different category of what appear as late responding genes. Formally, memory genes are defined as genes that respond to S1 by altering transcription but display different transcriptional responses in subsequent stresses. As these late responding genes do not appear among the genes that respond to the first stress, we have not included them in our analyses here. For each different response class the genes were separated into groups based on (inferred) GO terms (Additional files 5 and 6).

The raw transcriptome sequence files for watered, S1, and S3 have been uploaded, together with gene expression result files, to NCBI’s Gene Expression Omnibus under sequence number GSE48507.

## Results

### Dehydration stress responding memory and non-memory genes of *Zea mays*

**Figure 1:**
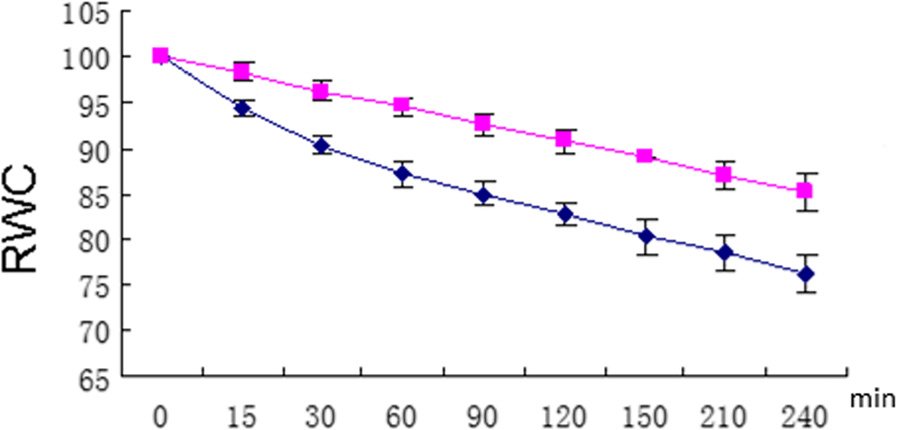
**Relative water content (RWC, %) in response to dehydration stress in leaves of trained or non-trained maize plants.** RWC was measured in leaves harvested after air drying for the indicated times (in min, shown on the ‘x’-axis), for plants experiencing their first stress (dark blue line) or for trained plants exposed to three stress cycles (pink line). Points are the mean, and error bars are ± SE from three independent experiments, each performed with 8–10 leaves from three separate plants.

Two-week-old maize seedlings were treated in the repetitive dehydration stress system developed in our laboratory for Arabidopsis [8]. Like Arabidopsis plants, previously stressed maize plants exhibited a reduced rate of water-loss upon a second exposure to dry air (Figure 1). This behavior, consistent with dehydration stress memory, allowed us to address the question of whether maize genes display transcriptional memory in a subsequent dehydration stress.

Genome-wide quantitative analysis of transcript levels of RNA samples isolated from seedling leaves of non-stressed watered (W), after a first dehydration stress (S1), and after the third exposure to dehydration stress (S3) (each stress following full 22 h watered recovery periods) were analyzed. Significantly different levels of transcripts in S1 compared to levels in W define the general dehydration stress response genes. Within this response fraction, genes that display significantly different transcript levels in S3 compared to S1 define the memory category, while genes that produce transcripts in S3 at comparable levels as in S1 comprise the non-memory category.

**Figure 2:**
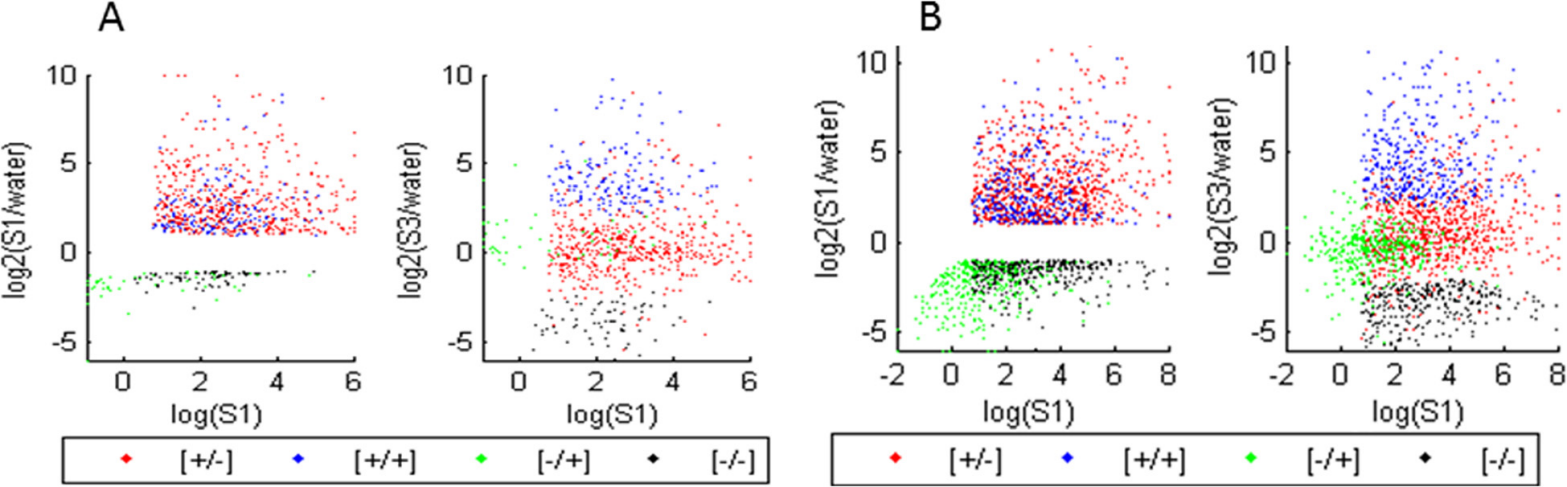
**Distribution of dehydration stress responding genes in Z. mays and *A. thaliana *in S1 and S3. A)** Transcript levels from dehydration stress responding genes that are up-regulated or down-regulated during S1 (color key at the bottom) plotted by the log2 of their S1 levels along the x-axis, and by log2 of their S1/watered ratio along the y-axis (left-hand panel); in the right-hand panel, transcript levels of dehydration stress memory genes are as above, except the y-axis is the log2 of the S3/watered ratio. The clustering of the four colors illustrates the distribution of the four distinct memory response types: revised response [+/-] and [-/+] memory genes clustering closer to their pre-stressed (W) levels, while the [+/+] and [-/-] increasing separation from these levels. **B)** Transcript levels from dehydration stress responding genes and from the memory genes in *A. thaliana*. Data are from Ding et al. 2013.

A total of 39,635 maize genes were identified (Additional file 3). Of these, 2,062 genes represented the general dehydration response fraction: 1,636 genes were upregulated and 426 were downregulated in S1 compared to levels in W (Table 1; Additional file 3). Within the dehydration response fraction, 816 genes (~40%) provided different responses in S3 constituting the maize transcription memory category, while 1246 genes produced similar transcript amounts upon each stress treatment representing maize ‘non-memory’ genes (Table 1; Additional file 3). Distribution of the dehydration stress responses of maize genes in S1 and in S3 is illustrated in Figure 2A.

**Table 1:** **Dehydration response and transcriptional memory genes in *Z. mays *** **and *A. thaliana***

		**Maize**	**Arabidopsis**
**Total genes by RNA-Seq**		39,635	33,555
**Dehydration response**		2,062	6,579
Induced		1,636	3,396
Repressed		426	3,183
**Non-memory genes**		1,246	4,616
Induced	[+/=] (W < S1 = S3)	941	2,177
Repressed	[-/=] (W > S1 = S3)	305	2,439
**Memory genes**		816	1,963
[+/+] (W < S1 < S3)		162	362
[-/-] (W > S1 > S3)		72	310
[+/-] (W < S1 > S3)		533	857
[-/+] (W > S1 < S3)		49	434
**Late-response genes**		2,924	1,371
[=/+] W = S1 < S3		1,678	798
[=/-] W = S1 > S3		1,246	573

### Memory-type responses by *Zea mays* dehydration stress responding genes

**Figure 3:**
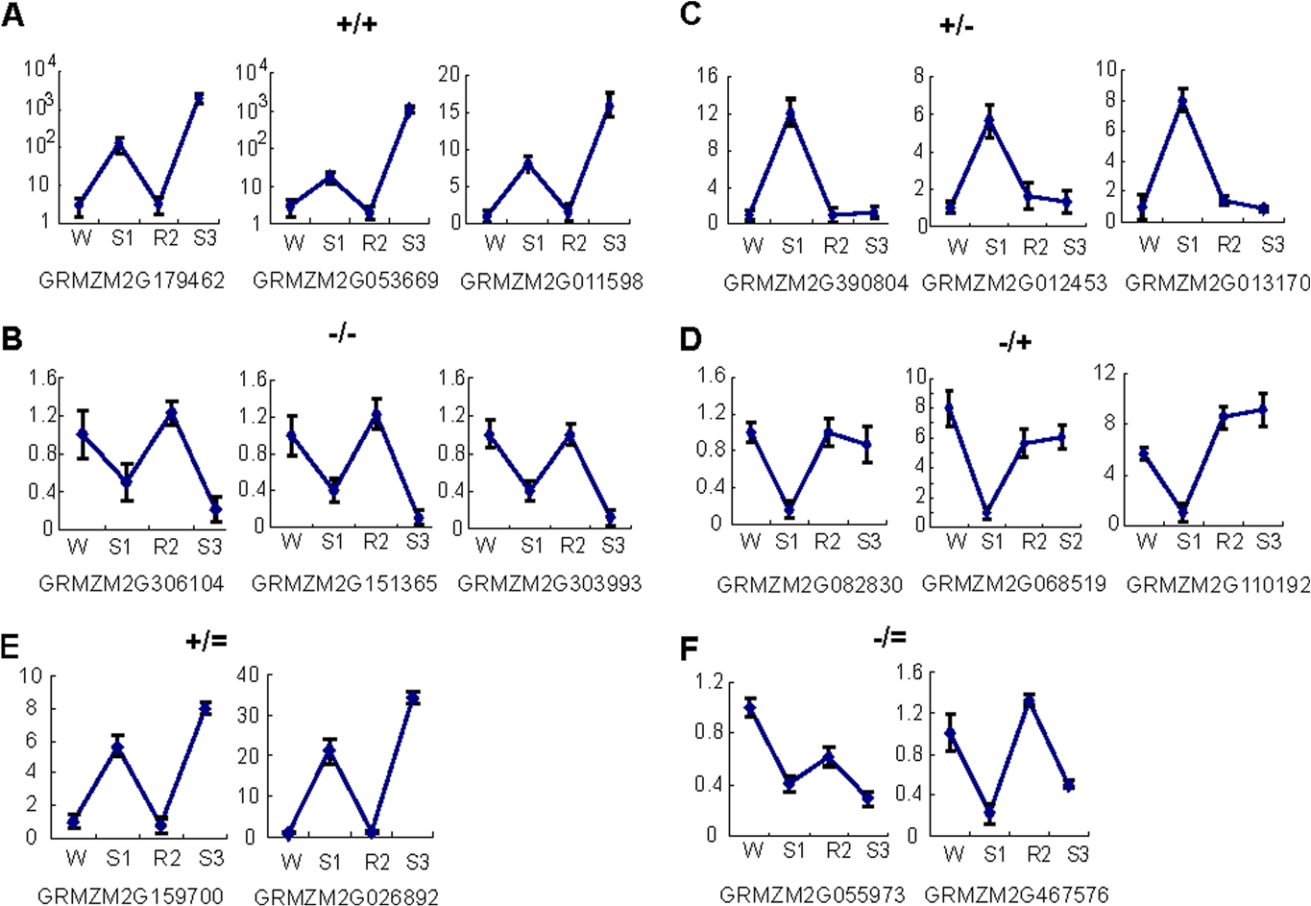
**Transcription patterns of maize dehydration stress responding genes under initial pre-stressed watered (W) conditions, upon the first exposure (S1), during watered recovery (R2), and upon a subsequent stress (S3) measured by real time qRT-PCR. A)** Higher transcript levels in S3 than in S1 produced by [+/+] memory genes: *GRMZM2G179462* encodes a putative low-temperature ABA-induced protein integral to the plasma membrane; *GRMZM2G053669* encodes a putative asparagine synthase; *GRMZM2G011598* encodes a NAC domain protein involved in hypersensitive response; **B)** lower transcripts in S3 than in S1 produced by [-/-] memory genes: *GRMZM2G306104* encodes a putative plastid gene; *GRMZM2G151365* encodes unknown protein; *GRMZM2G303993* encodes putative RNA-helicase; **C)** genes induced in S1 but revising response in S3 [+/-] memory genes: *GRMZM2G390804* encodes a protein with unknown function; *GRMZM2G012453* encodes a putative microtubule organizing protein; *GRMZM2G013170* encodes a putative disease resistance protein from the RPP family; **D)** genes repressed in S1 but regaining activity in R1 and maintaining it in S3 [-/+] memory genes: *GRMZM2G082830* encodes a putative membrane-associated kinase; *GRMZM2G068519* encodes a putative Flavin-binding oxygenase; *GRMZM2G110192* is an *NCED4* homolog; **E)** and **F)** genes repetitiously providing similar transcript amounts during each exposure to the dehydration stress: [+/=] *GRMZM2G159700* and *GRMZM2G026892* encoding a protein of the cupin-superfamily and an unknown protein, respectively. The [-/=] nontrainable genes *GRMZM2G055973* and *GRMZM2G467576* encode putative Zn-finger proteins from the RING family.

Comprehensive analyses of the transcriptome data revealed the existence of four distinct transcription patterns that were similar to the memory responses recognized earlier in *A. thaliana* ([9]; Additional file 4). Genes induced in S1 but superinduced in S3 are denoted as [+/+] memory genes (Figure 3A); genes with lower expression in S1 but producing transcripts at even lower levels in S3 represent the [-/-] memory type (Figure 3B).The highest number of maize genes with significant differences in S3/S1 transcript levels displayed increased transcript levels in S1, but decreased transcript levels in S3 relative to their S1 levels (the [+/-] memory type), Figure 3C. A complementary transcription pattern, displayed by genes with lower transcript levels in S1 but producing significantly higher transcript levels in S3, defines the [-/+] memory response category (Figure 3D). The unique feature of the latter two categories is that these genes ‘revise’ their transcription during a second exposure returning to their pre-stressed levels of transcription. In contrast, non-memory genes produce similar levels of transcripts in S1 and S3, (whether repetitively induced (Figure 3E), or reduced (Figure 3F) in response to each treatment.The distribution of the maize memory genes according to their transcription patterns in S3 (Figure 2A) clearly illustrate the clustering of the transcript levels of the revised response [+/-] and [-/+] memory genes closer to their pre-stressed (W) levels, while the [+/+] and [-/-] memory genes form clusters increasingly separated from the pre-stressed levels and from the levels in S1.

About 3,000 genes did not respond to the first stress, remaining at the initial pre-stressed levels but increased, or decreased, transcript levels during the subsequent (S3) stress. These genes annotated as [=/+] and [=/-] classes (included in Table 1 and Additional files 3, 4, 5, 6) are not analyzed further in this study as we focus on memory genes, which, according to our definition, belong in the gene fraction responding to S1.

### Functional distribution of *Zea mays* memory genes

To establish potential functions encoded by the memory response genes, the maize sequences were searched against annotated gene models for *A. thaliana.* The functional distribution within the memory categories is evaluated according to the Gene Ontology (GO) classification of the *A. thaliana* homologs. With the potential caveat that some genes might be annotated incorrectly in the GO database, maize memory genes were analyzed for their associated with various membranes, transcription regulatory functions, as well as for involvement in other abiotic or hormonally regulated pathways.

### Membrane-associated functions

**Figure 4:**
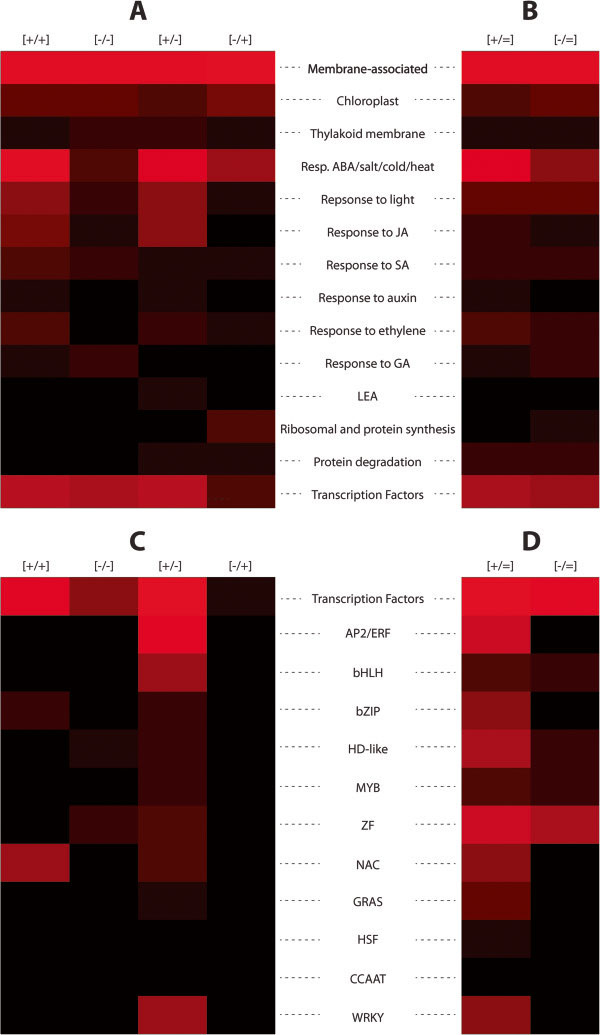
**Heat map illustrating the distribution of maize dehydration stress memory and non-memory classes according to different functional, biological process, and cellular component groups.** Heat maps follow the percentage-wise distributions of genes (Table 2, and Additional file 6) over various groups according to GO categories from the four memory classes **(A)** or the non-memory class **(B)**. Distribution of TFs encoded by memory genes **(C)** and non-memory genes **(D)***in Z. mays*. Pitch-black indicates 0%, and red is a linear increase in brightness to a maximum of 60% recalculated to follow the total number of transcription factor genes in the memory or non-memory categories. The *heatmap* function in MATLAB® was used to generate Figure 4.

The largest functional category, displayed by the entire dehydration stress responding maize transcriptome (accounting also for 22-36% of the genes from each memory subgroup) represents plasma and organelle membrane-associated proteins (Table 2; Additional file 6; Figure 4). The genes for membrane-associated functions constitute the largest fraction among the smallest [-/+] memory category (Table 2).

**Table 2:** Distribution of Maize memory-type genes according to GO functions

		**[ +/+ ]**	**[ -/- ]**	**[ +/- ]**	**[ -/+ ]**	**[ +/= ]**	**[ -/= ]**
		**162**	**72**	**533**	**49**	**941**	**305**
**Membrane-associated**^**a**^		42 (26%)^b^	16 (22%)	163 (31%)	18 (37%)	264 (28%)	86 (28%)
		[19%]	[23%]	[29%]	[28%]	[28%]	[23%]
**Chloroplast**		8 (5%)	4 (6%)	23 (4%)	3 (6%)	41 (4%)	15 (5%)
		[3%]	[24%]	[2%]	[5%]	[2%]	[8%]
**Thylakoid membrane**		2 (1%)	2 (3%)	12 (2%)	1 (2%)	12 (1%)	6 (2%)
		[0%]	[17%]	[0.7%]	[2%]	[0%]	[7%]
**RESP. Aba/Salt/Cold/Heat**		47 (29%)	3 (4%)	86 (16%)	4 (8%)	171 (18%)	22 (7%)
		[21%]	[10%]	[25%]	[10%]	[20%]	[7%]
**Response to light**		13 (8%)	2 (3%)	37 (7%)	1 (2%)	43 (5%)	14 (5%)
		[5%]	[8%]	[3%]	[6%]	[3%]	[4%]
**Response to JA**		10 (6%)	1 (1%)	37 (7%)	-	32 (3%)	6 (2%)
		[2%]	[5%]	[14%]	[2%]	[4%]	[2%]
**Response to SA**		7 (4%)	2 (3%)	11 (2%)	1 (2%)	30 (3%)	10 (3%)
		[1%]	[2%]	[5%]	[3%]	[3%]	[1%]
**Response to Auxin**		2 (1%)	-	11 (2%)	-	13 (1%)	2 (0.7%)
		[2%]	[2%]	[4%]	[1%]	[3%]	[2%]
**Response to ethylene**		7 (4%)	-	18 (3%)	1 (2%)	37 (4%)	8 (3%)
		[1%]	[1%]	[6%]	[2%]	[4%]	[1%]
**Response to GA**		3 (2%)	2 (3%)	6 (1%)	-	15 (2%)	8 (3%)
		[1%]	[2%]	[1%]	[1%]	[0%]	[0.7%]
**Lea**		-	-	8 (2%)	-	10 (1%)	-
		[3%]	[ND]	[0%]	[0%]	[0%]	[0%]
**Ribosomal and protein synthesis**		-	-	2 (0%)	2 (4%)	1 (0%)	5 (2%)
		[0%]	[10%]	[0%]	[ND]	[0%]	[5%]
**Protein degradation**		-	-	11 (2%)	1 (2%)	23 (2%)	7 (2%)
		[0%]	[0%]	[1%]	[0%]	[2%]	[0%]
**Transcription factors**		17 (11%)	7 (10%)	57 (11%)	2 (4%)	92 (10%)	27 (9%)
		[8]	[2%]	[7%]	[6%]	[7%]	[4%]
AP2/ERF		-	1 (14%)	17 (30%)	-	17 (19%)	1 (4%)
		[17%]	[17%]	[22%]	[8%]	[12%]	[10%]
bHLH		-	1 (14%)	8 (14%)	-	5 (5%)	3 (11%)
		[7%]	[17%]	[22%]	[8%]	[7%]	[16%]
bZIP		3 (18%)	-	3 (5%)	1 (50%)	10 (11%)	-
		[14%]	[ND]	[5%]	[8%]	[8%]	[1%]
HD-like		1 (6%)	2 (29%)	3 (5%)	-	12 (13%)	4 (15%)
		[10%]	[17%]	[8%]	[12%]	[16%]	[16%]
MYB		1 (6%)	-	3 (5%)	1 (50%)	5 (5%)	3 (11%)
		[14%]	[17%]	[11%]	[12%]	[10%]	[9%]
ZF		1 (6%)	3 (43%)	4 (7%)	-	17 (19%)	13 (48%)
		[10%]	[34%]	[8%]	[44%]	[25%]	[28%]
NAC		8 (47%)	-	4 (7%)	-	9 (10%)	-
		[3%]	[ND]	[10%]	[4%]	[8%]	[1%]
GRAS		-	-	2 (4%)	-	6 (7%)	1 (4%)
		[3%]	[ND]	[3%]	[ND]	[2%]	[6%]
HSF		1 (6%)	-	-	-	2 (2%)	1 (4%)
		[3%]	[ND]	[4%]	[ND]	[2%]	[ND]
CCAAT		1 (6%)	-	1 (2%)	-	-	-
		[10%]	[ND]	[0%]	[ND]	[1%]	[3%]
WRKY		1 (6%)	-	8 (14%)	-	9 (10%)	1 (4%)
		[7%]	[ND]	[7%]	[4%]	[9%]	[4%]

^a)^Membrane-associated include plasma membrane (PM), transmembrane (TM), kinases/receptors/signal transduction, TM transport, porins, and Wall/PM proteins

^b)^Number of genes and percentages (parentheses) per memory group and function are reported, with a comparison to the percentage of genes found in *Arabidopsis thaliana* [square brackets]. Only percentages equal or higher than 0.7 are reported, and higher than 1% are rounded to the nearest integer. For the *Arabidopsis* comparison, 0% means detected but smaller than 0.7%, while ND denotes Not Detected. The specific transcription factor subcategories (AP2/ERF, bZIP, etc.) show percentages based on the overall number of memory genes encoding transcription factors.

Memory genes associated with the thylakoid membrane, however, are low (1–2%) among all four memory categories, as well as among the non-memory genes repetitiously responding to each consecutive stress. It is noted that the highest numbers of genes encoding chloroplast and thylakoid membrane proteins respond to repeated dehydration stresses but not to the first stress (Additional files 5 and 6).

Despite their apparently even distribution, however, the membrane-associated proteins of each memory type cluster in distinct subgroups that differ by the nature of the metabolic processes they participate in, the specificity of the porin channels, the types of molecules transferred across membranes, and by the roles played in membrane protection, resistance against pathogens, disease and toxic metals (Additional file 6). For example, 26% of the [+/+] memory genes encode membrane-associated dehydrins, metabolic enzymes for synthesis of protective molecules, i.e., osmolytes, and for transmembrane transport of amino acids, oligopeptides, and carbohydrates; 22% of [-/-] memory genes encode membrane-associated kinases, receptor kinases, and signal transducers responding to light, transmembrane transporters for inorganic phosphate and sucrose; 36% of the [-/+] memory genes encode membrane-associated kinases, components of Ca^2+^- mediated signaling pathways and defense proteins, while 30% of the [+/-] memory genes encode water transport, K^+^ transport/uptake regulators, and a broad spectrum of proteins implicated in resistance to various metals, pathogens, and diseases (for full lists of GO function distribution among the memory types see Additional file 6).

### Dehydration stress memory genes shared with other abiotic-stress response pathways

Common genes co-regulated by different abiotic stresses or plant hormonal stimuli represent overlapping points between these networks and are considered central in coordinating the transcriptional responses to environmental factors [21-24]. Abscisic acid (ABA) is the key mediator of a large number of abiotic stress response systems [22,25]. About 400 maize ‘cross talk’ genes implicated in ABA, salt, cold, temperature, light responses, were induced and ~50 genes were repressed during the first stress (Additional file 6). Upon subsequent treatments, however, these S1-responding genes provided responses segregating into the six different transcriptional patterns. Most of these genes continued to respond similarly to each stress, represented by ~200 [+/=] and 36 [-/=] non-memory genes. Among the memory cross talk genes, there were 60 [+/+] and 5 [-/-] response genes. However, 128 revised response genes (from both [+/-] and [-/+] categories) displayed responses in S3 as in W, virtually ‘cancelling’ the crosstalk with the other response pathways triggered in S1. Of note are the much lower numbers of cross talk genes that have decreased transcription during the first stress (46 [-/-], [-/+] and [-/=] response genes) compared to the number of cross talk genes that displayed increased transcript levels during a first stress (397 [+/+], [+/-] and [+/=]) (Table 2; Figure 4; Additional file 6).

### Dehydration stress memory genes shared with hormonally regulated pathways

Dehydration stress memory responsive genes overlap with genes regulated by hormone signaling pathways, although in much lower numbers than those shared with abiotic stress-responding genes (Table 2; Figure 4; Additional file 6). Most of the shared genes induced in S1 were implicated in ABA, ethylene, JA, and SA-responses, an observation consistent with synergistic interactions between these pathways under drought [21,26,27]. Jasmonate (MJ), considered the most important signal in biotic stress responses and wounding, works both cooperatively and antagonistically with ABA during dehydration stress [28-30]. Our data identified 86 co-regulated genes that altered expression under the first dehydration stress: 79 shared genes increased and seven genes decreased transcription (Table 2; Additional file 6). Upon a subsequent exposure, however, these shared genes provided diverse responses: 32 [+/=] and six [-/=] genes continued to produce similar transcript levels, ten produced transcripts at higher [+/+], one at lower [-/-] levels, and 37 genes did not respond. These latter common [+/-] memory dehydration/JA responding genes elevate transcription during the first encounter with the dehydration stress but upon a second exposure are transcribed as in their non-stressed state and, therefore, do not provide a transcriptional response to the subsequent dehydration stresses. Similar segregation of the transcription patterns during repeated exposures to dehydration stress were observed also for genes shared with the other plant hormones (Table 2; Additional file 6; Figure 4).

### Memory genes encoding Transcription Factors (TFs) in *Zea mays*

Only a limited number of TF families are represented by dehydration stress memory genes in *Z. mays,* with a strongly biased distribution among the four memory categories (Table 2; Figure 4; Additional file 6): 83 maize genes (~10% of the maize dehydration stress memory group) encode TFs represented almost exclusively by [+/+] and [+/-] memory genes, while only nine TFs genes were downregulated during the response to the first stress (seven [-/-] and two [-/+] memory genes, Table 2; Additional file 6). Most TF families are represented by one to four genes from each memory class except the *NAC* family TF genes, which stand out as a signature [+/+] memory TF family; genes from the *AP2/ERF* (Integrase-type) family, from the *bHLH* and *WRKY* families are found exclusively among the [+/-] memory subgroup. Interestingly, [+/+] memory *NAC* family genes are shared with the ABA, ethylene, JA, SA and abiotic stress responding, while [+/-] *Integrase-type AP2/ERF* family memory genes are shared predominantly with ABA and ethylene, but not the JA and SA, pathways. The *bHLH* and *WRKY* family members are implicated in responses to pathogens, metal ions, JA and SA (Additional file 6).

### Comparing *Zea mays* and *Arabidopsis thaliana* dehydration stress responding genes

Overall, a total of 39,635 maize genes, of which 2,062 genes (~4.5% of the maize transcriptome) significantly altering transcription in S1 compared to W were identified by RNA-seq transcriptome analyses (Table 1; Additional file 3). By comparison, 6,597 genes among 33,555 genes identified in *A. thaliana* (~20% of its transcriptome) were involved in the first dehydration stress response (Table 1; [9]). The data underscore a remarkable difference in the numbers of genes implicated in the responses to the first dehydration stress at the transcriptional level in the two species, which is also illustrated by the density of the clouds in Figure 2A and Figure 2B.

Other species-specific differences in the transcriptional responses to the first stress were the numbers of *Z. mays* genes induced in S1 outnumbering downregulated genes in a ratio 4:1, while in *A. thaliana* the numbers are comparable (Table 1; [9]). The paucity of downregulated maize gene in response to the first stress is illustrated in Figure 2A. Furthermore, although a smaller number of genes is implicated in the dehydration response in maize, the 816 genes displaying memory behavior constitute almost 40% of the entire *Z. mays* fraction responding in S1, while memory genes constitute ~30% of the dehydration response fraction of *A. thaliana* (Table 1).

### The memory genes of *Zea mays* and *Arabidopsis thaliana*

One of the main goals of this study was to find out whether transcriptional memory responses were conserved in the two species. Structurally related genes that display the same transcriptional memory in the two species would suggest conservation of memory during the evolution of monocot and eudicot dehydration stress response systems. On the other hand, divergent transcriptional responses by genes encoding similar functions would suggest memory behavior reflecting species-specific responses to repeated exposures to dehydration stress.

A search with the 816 *Z. mays* memory genes against the entire *A. thaliana* genome sequences identified 2,284 homologous sequences (above threshold levels defined in Methods), referred to from hereon, as Arabidopsis homologs. The higher number of *A. thaliana* genes homologous to the *Z. mays* memory genes reflects the fact that more than one family member gene satisfy the sequence similarity threshold (Additional file 4).

**Figure 5:**
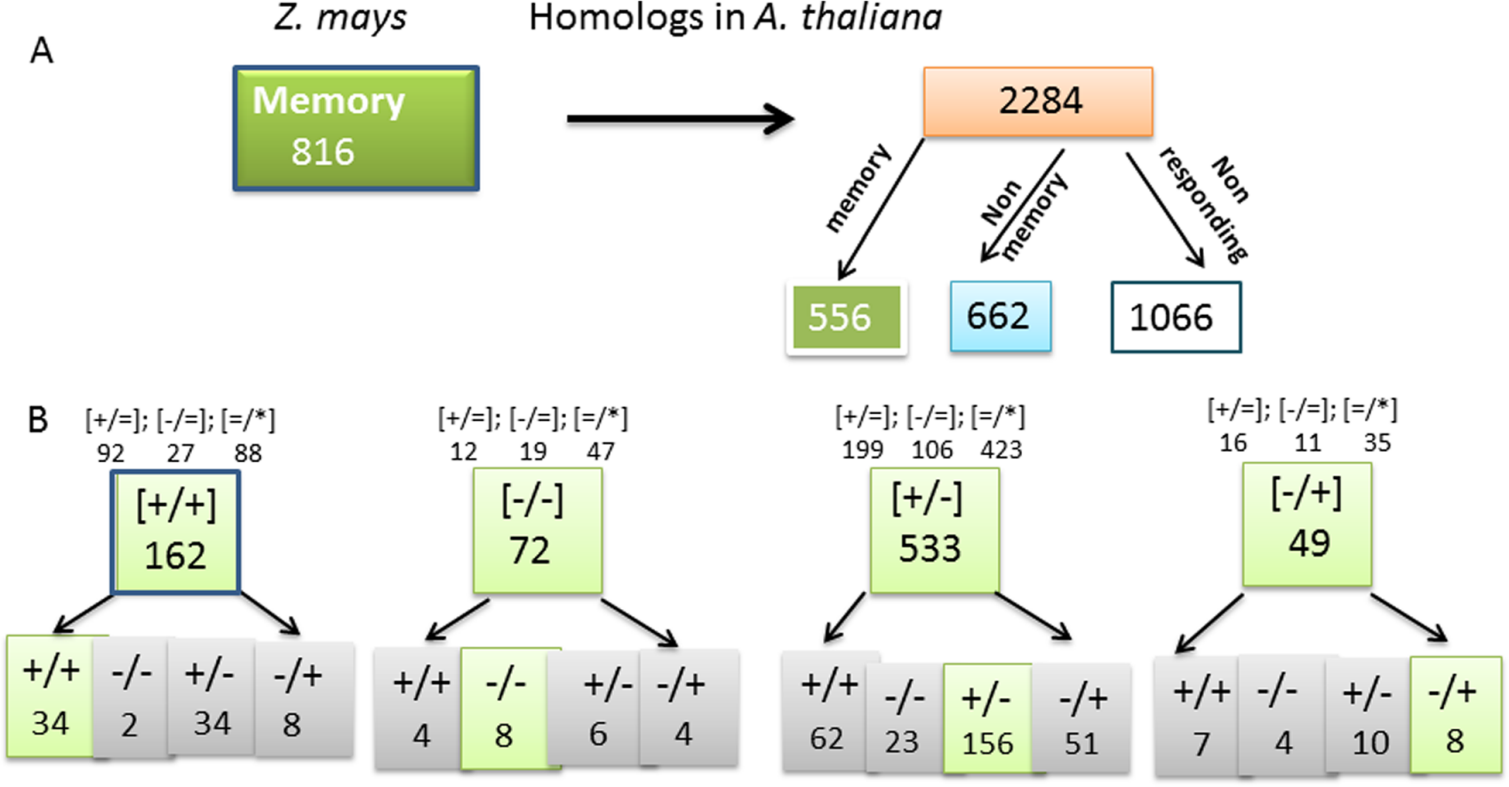
**Distribution of the response patterns to repeated dehydration stress by homologous genes in the two species. A)** Overall maize memory genes and their homologs in Arabidopsis are shown in the larger frames. The numbers of Arabidopsis homologous genes distributed according to the patterns of their responses to repeated dehydration stress are in the smaller frames below. Comparable numbers of the homologs respond to dehydration stress by providing memory or non-memory responses in Arabidopsis. The largest numbers of homologous genes not belonging in the dehydration response category are indicated. Members of gene families homologous to a maize memory gene account for the higher numbers of genes in Arabidopsis. **B)** Distribution of the maize homologs in Arabidopsis according to the memory response type. The numbers of maize genes of a particular memory category are in larger frames. The numbers of respective homologs according to their responses in Arabidopsis are in smaller frames: memory response genes are in shaded brackets (bottom rows); conserved-memory responses are shaded in green; non-memory genes and Arabidopsis homologs that do not respond in S1 (indicated by the =/* sign) are in brackets on top. Multiple members with a gene family responding differently to dehydration stress account for the higher numbers of summed up genes in both species.

Almost half (1,066 genes) of the *A. thaliana* homologs showed no change in transcript levels in S1 and, therefore, do not belong in the general dehydration stress response fraction; about half of the remaining *A. thaliana* homologs (662 genes) respond to dehydration stress but lack memory, while 556 are dehydration stress memory genes in Arabidopsis as well (Figure 5A; Additional file 6).

Whether the homologs of the *Z. mays* memory genes display similar types of memory responses in *A. thaliana* was investigated next. The *Z. mays* genes from each memory category were compared with the responses of their respective homologs in Arabidopsis (data are from reference [9]). The results from the analysis indicated that the Arabidopsis homologs of the maize memory genes displayed a wide spectrum of responses including conserved and non-conserved memory responses, but homologs that lack memory or were not involved in the first dehydration stress response were the largest categories (Figure 5B; full lists in Additional file 7).

The memory genes of *Z. mays* that have homologs with the same memory-type behavior in *A. thaliana* occur in all four memory response types but differ in the numbers: 34 [+/+], eight [-/-], eight [-/+], and 156 revised response [+/-] maize memory genes have homologs that carry the same transcriptional memories in Arabidopsis (Figure 5B). In addition, homologous genes display memory responses that are of different transcription memory types in the two species (Figure 5B, full lists in Additional file 7). These genes, in addition to being conserved as dehydration stress responding genes in maize and Arabidopsis, have conserved also their ability to provide memory responses to repeated stress. However, the different memory patterns displayed by the homologous genes suggest species-specific diversification of the responses to repeated stress occurring at the transcriptional level during their evolution.

### Functional distribution of *Z. mays* and *A. thaliana* memory genes

Assuming that structurally similar genes in the two species have similar cellular functions, we performed comparative analysis of the functional distribution within the memory categories based on the Gene Ontology (GO) classification of the *A. thaliana* homologs. The numbers of maize genes and their percentages per memory group are reported in Table 2. The percentages per memory group and GO function shown for *A. thaliana* genes are from [9].

**Figure 6:**
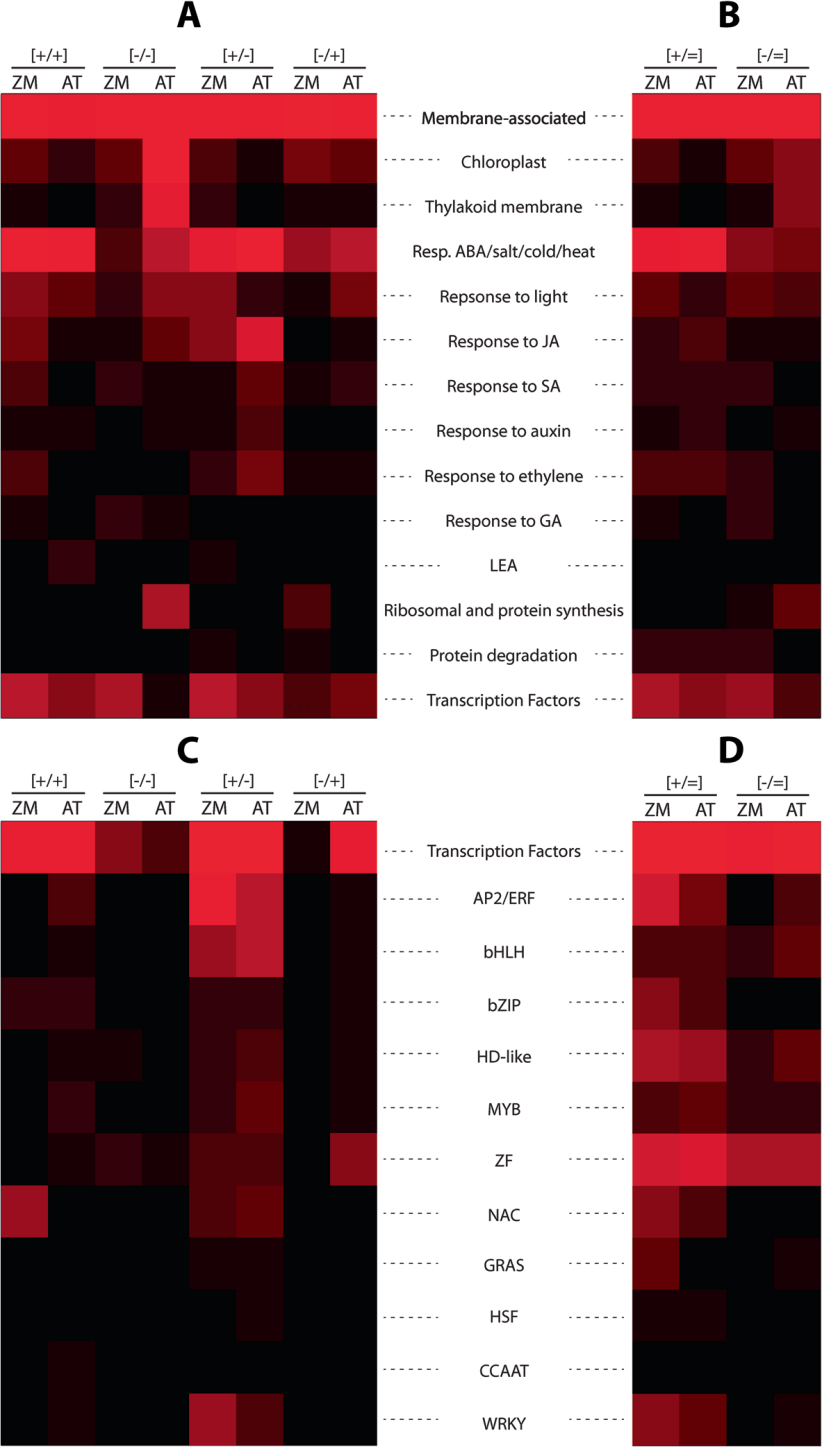
**Pair-wise comparison of *Z. mays *and *A. thaliana *memory and non-memory genes according to different functional, biological process, and cellular component groups.** Heat maps follow the percentage-wise distributions of the genes (Table 2, Additional file 7) over various groups according to GO categories from the four memory classes **(A)** or the non-memory class **(B)** for *Z. mays* (ZM) and *A. thaliana* (AT). Distribution of TFs encoded by memory genes **(C)** and non-memory genes **(D)** in *Z. mays* and *A. thaliana*. Pitch-black indicates 0%, and red is a linear increase in brightness to a maximum of 60% recalculated to follow the total number of transcription factor genes in the memory or non-memory categories. The *heatmap* function in MATLAB® was used to generate Figure 6.

Comparison of the cellular functions encoded by the memory genes in the two species revealed some remarkable differences. Although the largest numbers of memory genes encode membrane-bound functions in both species, it is noted that *Z. mays* proteins associated with chloroplast and thylakoid membrane functions are encoded by a very low number of [-/-] memory genes (four chloroplast and two thylakoid membrane genes, respectively), in a stark contrast with the 128 *A. thaliana* [-/-] memory genes implicated in these functions (Table 2, Figure 6). *Z. mays* genes implicated in abiotic stress responses and in ribosome organization/protein synthesis, are also represented by much lower numbers of the [-/-] and [-/+] memory genes than in *A. thaliana* (Table 2, Figure 6).

Species-specific distributions of the dehydration stress/JA co-regulated genes among the [+/+] and [+/-] memory genes (Table 2; Figure 6) and the memory genes encoding TFs in *Z. mays* and *A. thaliana* are displayed as well. Although similar percentage (~10%) of memory genes encode TFs, the TF family types and their distribution among the memory categories is species-specific. Given the limited number of TF families represented by 1–3 genes per family in *Z. mays* (Table 2; Additional file 7), the [+/+] memory genes encoding NAC family TFs (amounting to ~50% of all TFs from the [+/+] memory category) and the [+/-] memory genes encoding Integrase-type AP2/ERF family, are standing out as exclusive features of the *Z. mays* TF memory genes. Only two [-/+] memory genes encoding TFs were identified in maize (Additional file 7).

## Discussion

### Biological relevance of *Z. mays* dehydration stress memory genes

The majority of the 816 genes of the transcription memory fraction in *Z. mays* belong in gene families containing other members, which may display diverse memory or non-memory responses. For example, ten maize *PP2C*-class phosphatase genes are strongly induced in S1. Upon repeated treatments, however, three genes (*GRMZM2G159811, GRMZM2G308615, GRMZM5G818101*) elevate transcription further ([+/+] memory genes), two are not induced by the second treatment (the [+/-] memory genes *GRMZM2G465287, GRMZM2G342197*), and five [+/=] genes (*GRMZM2G010855, GRMZM2G059453, GRMZM2G166297, AC208201.3_FG002, GRMZM2G000603*) repetitiously produce transcripts at S1 levels. As PP2C phosphatases are essential in the ABA mediated signaling [31-33], the transcriptional responses of individual *PP2C* genes under repeated exposures is likely to affect specifically and differently the behavior of the ABA-signaling and other overlapping networks during multiple encounters with dehydration stress.

Presumably, the behavior of dehydration memory genes allows plants to modulate their transcriptional responses during repeated stresses to improve survival. Increased transcripts from [+/+] memory genes encoding protective functions (dehydrins, heat shock proteins, chaperones) and diverse metabolic enzymes involved in detoxification, in the synthesis of osmolytes and membrane-protecting lipids are consistent with the general strategy employed by plants during stress [31]. In addition, maintenance/re-adjustment of cellular homeostasis and of the interactions between dehydration and other stress/hormone regulated pathways are considered critical for the survival [6,31,32]. It is logical to consider the biological relevance of [-/+] and [+/-] memory genes in this context, as suggested by the large numbers of metabolic functions encoded by revised response memory genes (Additional file 6). After returning close to pre-stressed levels in S3, [+/-] and [-/+] memory genes implicated in various metabolic process may play roles in re-setting cellular metabolism, photosynthesis and energy balance to adjust to the new conditions.

Membrane-associated genes regulating osmotic pressure, water balance, and wall modifications are important in plants’ stress responses and environmental adaptation [33]. Genes encoding membrane-associated functions are encoded by all four memory categories in maize. Importantly, the distribution by the nature of membrane function among the memory types is specific. Thus, membrane-associated proteins with protective functions (dehydrins) or for the synthesis of osmolytes are encoded by [+/+] memory genes (see further below), membrane-associated kinases, receptor kinases, and signal transducers responding to light, transmembrane transporters for inorganic phosphate and sucrose are encoded by [-/-] and [-/+] memory genes, while [+/-] memory genes encode water transport, ion transport/uptake regulators, and proteins implicated in resistance to various metals, pathogens, and diseases (for full lists of GO function distribution among the memory types see Additional file 6).

We note also the [+/-] memory genes co-regulated with other abiotic stresses or hormonally regulated pathways. These transcriptional patterns revealed a much higher level of complexity of the interactions between these signaling pathways than displayed during a single dehydration stress. By altering transcription, shared memory genes are likely to affect the crosstalk during subsequent stresses. Thus, shared [+/+] memory genes are producing more, [-/-] memory genes are producing fewer transcripts, while revised-response memory genes return transcripts closer to their initial (W) levels. Consequently, the crosstalk between dehydration and other stress response/signaling pathways will be different under repeated dehydration stresses from the crosstalk occurring during the first encounter. The crosstalk will be different also from the crosstalk of shared non-memory [+/=], [-/=] genes, which provide similar interactions during each stress (Table 2, Additional file 6). These modified responses may help the plant spare its resources when enduring repeated dehydration stress [6,34].

### Functions encoded by conserved memory genes in *Z. mays* and *A. thaliana*

Homologous genes with conserved [+/+] and [+/-] memory behavior in the two species encode proteins with cell protective functions and proteins implicated in readjustment of the metabolism: in *Z. mays*, [+/+] memory genes encode the dehydrin GRMZM2G079440, related to RAB18 and XERO1 in *A. thaliana*; cytochromes GRMZM5G851862 and GRMZM2G126505, involved in ABA metabolism and signaling are related to CYP76C2 and CYP707A3, respectively; enzymes for the biosynthesis of proline (GRMZM2G028535, homologous to P5CS1), for detoxification (GRMZM2G059836, GRMZM2G088396), and for TFs involved in regulation of various shared stress responding pathways (specific functions of all homologs according to a memory category are in Additional File 7). Conserved [+/-] memory homologs are implicated in hormone-regulated pathways (JA-signaling, in particular), encode TFs (predominantly from the AP2/ERF family), and diverse metabolic functions (Additional file 7). The function of 36 homologous [+/-] memory genes in Arabidopsis is unknown.

The species-specific distributions of the memory genes encoding TFs in *Z. mays* and *A. thaliana* are noted. Although similar percentages (~10%) of memory genes encode TFs in the two species, about half of the maize TF memory genes encode NAC family members, 30% encode Integrase-type AP2/ERF family members and 14% encode factors from the WRKY family (Table 2; Figure 6C). The representation of these family members among the memory genes of *A. thaliana* is remarkably different (see numbers in square brackets in Table 2). Revealing the roles of the dehydration memory transcription factor genes in the memory behavior of dependent genes will be of paramount significance for understanding the molecular mechanisms regulating transcriptional memory. It is emphasized, however, that the memory behavior of a TF does not necessarily determine the memory behavior of all of its targets even when the TF binds directly to the gene’s promoter. For example, the memory pattern of the *A. thaliana MYC2* gene is responsible for the transcriptional memory behavior of only a specific subset of the MYC2-dependent genes, while *RD22*, a marker MYC2-regulated gene [35], displays in S2 a response different from the memory response of MYC2 [36]. Therefore, the transcriptional behavior of a TF cannot be used to explain, or predict, the transcriptional behavior under repeated stresses of its target genes. Diverse and gene-specific mechanisms regulate transcription memory behavior of dehydration stress genes even for genes belonging in the same memory category [37].

Homologous proteins with similar protective/re-adjusting functions encoded by memory genes in one species may be encoded by genes that display non-conserved responses in the other. For example, in Arabidopsis the lipid transfer proteins (LTP2, LTP3, LTP4) and LEA proteins (implicated in protecting cell membrane structure, fluidity, and ion balance [34,38,39] are encoded by some of the most highly superinduced [+/+] memory genes; however, in maize, six LEA proteins are encoded by [+/-] memory genes, while the gene for GRMZM2G010868, homologous to LTP2/3/4 proteins, does not respond to the stress in S1; reciprocally, the maize [+/+] memory gene encoding a putative cold and salt responsive protein (GRMZM2G179462) implicated in ABA/abiotic stress responses has four gene homologs in the RCI2A family, all of which show non-memory (+/=) responses in Arabidopsis (Table 2; Figure 6; Additional file 7).

We suggest that structurally related homologous genes that display non-conserved memory responses indicate species-specific features reflecting divergence of the roles played by homologous proteins in the two species. Thus, seven genes involved in JA-signaling (the *JAZ* genes) and JA-responding genes have conserved [+/-] memory responses in both maize and Arabidopsis (Additional file 7). However, the *AOC* (allene oxide cyclase) genes critical for the biosynthesis of JA display [+/-] memory responses only in Arabidopsis; there are four *AOC* genes in maize, but only one provides a weak [+/=] non-memory response and three are not affected, suggesting a different crosstalk with the JA-pathway during dehydration stress in the two species.

### Evolution of the transcriptional memory

The highly sensitive RNA-seq transcriptome analyses allowed identification of genes that function similarly in the two lineages as well as genes that function in species-specific ways. By modifying their transcriptional responses, the memory genes are likely to finely tune synthesis of protective cellular functions, to modify interactions with other signaling networks, and to re-adjust physiological processes under repeated cycles of dehydration stress. Of particular note is that some homologs of the maize memory genes behave as memory genes in Arabidopsis but display different transcriptional response patterns (Figure 5B; Additional file 7). Thereby, although some evolutionarily conserved genes involved in dehydration stress response have been conserved as memory genes in the two species, they belong in different memory response categories. This may reflect specific tuning and adjusting of cellular functions employed by a C4 monocot and a C3 eudicot plant in response to repeated dehydration stresses.

The most dramatic differences between the maize and Arabidopsis memory responses to multiple dehydration stresses are displayed by the [-/-] and [-/+] memory genes. Thus, only eight [-/-] genes and eight [-/+] genes in maize have homologs with the same memory responses in Arabidopsis (Figure 5B; Additional file 7). In a stark contrast with the large number of chloroplast and thylakoid membrane [-/-] memory genes in Arabidopsis, only four chloroplast and two thylakoid membrane genes display [-/-] memory genes in maize and none of the conserved [-/-] or [-/+] memory genes between the two species encodes a chloroplast/thylakoid membrane-related function (Table 2; Figure 6; Additional files 6 and 7). Together, these memory responses by the homologous genes of maize and Arabidopsis suggest significant differences in photosynthetic and related metabolic functions employed by two species (with C4 and C3 photosynthetic pathways, respectively) when experiencing repeated dehydration stresses.

## Conclusions

The most important result of this study is the evidence that a monocot and a dicot plant display dehydration stress memory and modify their transcriptional responses by similar transcriptional memory patterns. The RNA-Seq transcriptome analyses allowed identification of genes that function similarly in the two lineages, as well as genes that function in species-specific ways. The four memory transcription patterns indicate that the transcriptional behavior of dehydration stress responding genes under repeated stresses is more complicated than the behavior involved in a single dehydration stress, suggesting that dehydration stress memory is a complex phenotype resulting from coordinated responses of multiple signaling pathways. Despite evidence of conservation between the two lineages in terms of the presence of dehydration stress-responding genes, species-specific differences in the transcriptional responses were found. *First,* the number of *Z. mays* genes involved in the general (S1) dehydration stress responses (~4.5% of the maize transcriptome) is significantly lower than in Arabidopsis (~20% of its transcriptome). *Second,* induced maize genes outnumber repressed in a ratio 4:1, while in *A. thaliana* the numbers are comparable. *Third*, the memory genes in maize constitute almost 40% of the overall dehydration responding fraction, while in Arabidopsis the memory genes are about 30%, despite the much smaller number of maize genes implicated in the dehydration response than in Arabidopsis. *Fourth*, conserved homologous genes in the two species that display transcriptional memory but in different memory categories illustrate specific modifications of the memory responses during the evolution of the response systems under repeated dehydration stress occurrences. The results contribute to our current knowledge of how plants respond to multiple dehydration stresses and provide a reference platform for studies of the transcriptional responses to water deficit by monocot and eudicot plants.

## Competing interests

The authors declare that they have no competing interests.

## Authors’ contributions

LV, YD, and NL performed experiments. J-JR performed bioinformatics analyses. MF and ZA conceived the study and interpreted results. All authors read and approved the final manuscript.

## Supplementary Material

Additional file 1 Distribution of raw and mapped reads over samples and replicates. Click here for file

Additional file 2 Primers used in the qRT-PCR experiments. Click here for file

Additional file 3 **Full list of *Z. mays *** **genes identified on *A. thaliana *** **genes as models.** Click here for file

Additional file 4 **Transcript abundances displayed by the memory genes from the four memory categories and for the induced and repressed non-memory genes of *Z. mays.***
Click here for file

Additional file 5 Table of [=/+] and [=/-] response genes according to GO function. Click here for file

Additional file 6 Distribution of memory and non-memory genes according to selected GO categories. Click here for file

Additional file 7 ***Z. mays *** **memory genes and their homologs in *A. thaliana.***
Click here for file
