# Supplementary material for: Dehydration stress memory genes of Zea mays; comparison with Arabidopsis thaliana
Source: BMC Plant Biol. 2014 May 22;14:141. doi: 10.1186/1471-2229-14-141 (PMC4081654; doi:10.1186/1471-2229-14-141)
Supplement: Additional file 5 — Table of [=/+] and [=/-] response genes according to GO function. [file 1471-2229-14-141-S5.docx]

**Supplementary Table 5** Distribution of Maize Late-response genes according to GO functions

|  | | [ =/+ ] 1678 | [=/-] 1246 |
| --- | --- | --- | --- |
| Membrane-associated^a^ | | 489 (29%)^b^ [23%] | 269 (22%) [26%] |
| Chloroplast | | 77 (5%) [3%] | 156 (13%) [15%] |
| Thylakoid membrane | | 30 (2%) [1%] | 124 (10%) [4%] |
| resp. ABA/salt/cold/heat | | 157 (9%) [14%] | 86 (7%) [16%] |
| Response to light | | 60 (4%) [6%] | 54 (4%) [5%] |
| Response to JA | | 34 (2%) [1%] | 25 (2%) [4%] |
| Response to SA | | 35 (2%) [1%] | 27 (2%) [2%] |
| Response to auxin | | 16 (1%) [1%] | 12 (1%) [2%] |
| Response to ethylene | | 33 (2%) [2%] | 21 (2%) [1%] |
| Response to GA | | 23 (1%) [1%] | 20 (2%) [1%] |
| LEA | | 9 (0%) [0.7%] | 2 (0%) [ND] |
| RiBOsomal and protein synthesis | | 11 (0%) [0%] | 33 (3%) [5%] |
| Protein degradation | | 25 (1%)  [1%] | 17 (1%) [0.9%] |
| Transcription Factors | | 117 (7%) [2%] | 67 (5%) [4%] |
|  | AP2/ERF | 10 (9%) [2%] | 3 (5%) [14%] |
|  | bHLH | 18 (15%)  [10%] | 13 (19%) [ND] |
|  | bZIP | 22 (19%)  [6%] | 4 (6%) [ND] |
|  | HD-like | 12 (10%)  [16%] | 8 (12%) [14%] |
|  | MYB | 13 (11%)  [20%] | 7 (10%) [18%] |
|  | ZF | 29 (25%) [24%] | 22 (33%) [18%] |
|  | NAC | 8 (7%) [6%] | 1 (2%) [ND] |
|  | GRAS | - [2%] | 1 (2%) [9%] |
|  | HSF | 1 (1%) [4%] | 1 (2%) [5%] |
|  | CCAAT | 2 (2%) [4%] | 1 (2%) [ND] |
|  | WRKY | 2 (2%) [2%] | 6 (9%) [5%] |

^A)^ Membrane-associated include transmembrane (TM), kinases/receptors/signal transduction, TM transport, porins, and Wall/PM.

^b)^Number of genes and percentages (parentheses) per memory group and function are reported, with a comparison to the percentage of genes found in *Arabidopsis thaliana* [square brackets]. Only percentages equal or higher than 0.7 are reported, and higher than 1% are rounded to the nearest integer. For the *Arabidopsis* comparison, 0% means detected but smaller than 0.7%, while ND denotes Not Detected. The transcription factor (AP2/ERF, bZIP, etc) subcategories show percentages based on the main transcription factor category.
